# Spectroscopic Characterization and Biological Effects of 1-Oxo-bisabolone-rich *Pulicaria burchardii* Hutch. subsp. *burchardii* Essential Oil Against Viruses, Bacteria, and Spore Germination

**DOI:** 10.3390/plants14010068

**Published:** 2024-12-29

**Authors:** Giusy Castagliuolo, Federica Dell’Annunziata, Sara Pio, Michela Di Napoli, Alessia Troiano, Dario Antonini, Natale Badalamenti, Maurizio Bruno, Vincenzo Ilardi, Veronica Folliero, Mario Varcamonti, Gianluigi Franci, Anna Zanfardino

**Affiliations:** 1 Department of Biology, University of Naples, 80126 Naples, Italy; giusy.castagliuolo@unina.it (G.C.); sa.pio@studenti.unina.it (S.P.); michela.dinapoli@unina.it (M.D.N.); alessia.troiano2@studenti.unina.it (A.T.); dario.antonini@unina.it (D.A.); varcamon@unina.it (M.V.); anna.zanfardino@unina.it (A.Z.); 2 Department of Medicine, Surgery and Dentistry, Scuola Medica Salernitana, University of Salerno, 84081 Baronissi, Italy; federica.dellannunziata@unicampania.it; 3 Department of Experimental Medicine, University of Campania Luigi Vanvitelli, 84081 Naples, Italy; 4 Department of Biological, Chemical and Pharmaceutical Sciences and Technologies (STEBICEF), University of Palermo, Viale delle Scienze, 90128 Palermo, Italy; vincenzo.ilardi@unipa.it; 5 NBFC-National Biodiversity Future Center, 90133 Palermo, Italy; 6 UOS Microbiology and Virology, AOU San Giovanni di Dio e Ruggi d’Aragona, 84131 Salerno, Italy; vfolliero@unisa.it (V.F.); gfranci@unisa.it (G.F.)

**Keywords:** *Pulicaria burchardii* Hutch. subsp. *burchardii*, 1-oxo-bisabolone, antimicrobial activity, anti-germinative properties, HSV-1, SARS-CoV-2

## Abstract

*Pulicaria* species are used as herbal medicine and in the preparation of decoctions in several Asian and African regions. Among them, the plant *Pulicaria burchardii* is known for its medicinal properties, but comprehensive studies on its biological activity are still limited. This study examined the properties of the essential oil (EO) extracted by *P. burchiardii* and collected in Morocco during the flowering period. The focus was on its antimicrobial, anti-germinative, antioxidant, and antiviral activities, with the aim of evaluating its potential use in food preservation and beyond. The EO was subjected to various analyses to determine its chemical composition and biological efficacy. Specifically, GCMS and NMR analyses revealed that the EO is rich in oxygenated sesquiterpenes (72.59%), with 1-oxo-bisabolone being the predominant component (65.09%). The antimicrobial activity was tested against various Gram-positive and Gram-negative bacteria, demonstrating a significant inhibition of bacterial growth, particularly against *Bacillus subtilis* (MIC value of 0.6 mg/mL). The anti-germinative property was evaluated on spores of *B. subtilis* and other bacilli, such as *Bacillus cereus*, revealing a notable ability to prevent germination. For antiviral activity, the EO was tested against several pathogenic viruses including SARS-CoV-2 and HSV-1, showing an effective broad-spectrum reduction in viral replication in vitro. This study demonstrated that *P. burchardii* essential oil had excellent antibacterial and antiviral capabilities. The future challenge will focus mainly on the principal compound, 1-oxo-bisabolone, to demonstrate its real effectiveness as an antibacterial and/or antiviral.

## 1. Introduction

The *Pulicaria* genus (Asteraceae) includes eighty-two accepted species distributed in three different continents, Africa, Asia, and Europe, but the species are mainly concentrated in the Mediterranean basin region [1].

Interesting biological properties, such as cytotoxic [2], antibacterial [3], anti-inflammatory [4], antihistaminic [5], antifungal [6], insecticide [7], and leishmanicidal [8], have been assessed for several *Pulicaria* species.

*Pulicaria* species are commonly employed in folk medicine. In fact, the biological potential of some species such as *P. cruncha* and *P. incisa* used in the culinary field as drinks [9,10,11] or as food flavorings has been reported [11]. Some of these are also exploited to intensify the flavor or aroma of an infusion such as *P. undulata* [11] or *P. jaubertii* E. Gamal–Eldin [12,13]. *Inula arabica*, synonymous with *P. arabica* (L.) Cass, in turn, is used to relieve swelling and painful boils [14]. Other *Pulicaria* species, known in Iran as “kak kosh” and “shebang”, are commonly utilized as herbal teas, flavoring agents, and ethnobotanical remedies for inflammatory diseases and to treat severe heatstroke and diarrhea [15]. *P. odora* L. is a Moroccan medicinal, locally known as “Ouden El hallouf”; its properties, as traditional medicine, includes the treatment of back pain, cramps, and menstrual pain [16].

Different secondary products such as phenolic compounds, terpenoids with different skeletons, diterpenes, triterpenoids, have been identified in several *Pulicaria* species [17]. Their EOs, extracted from leaves, fruits, and even barks, have also been tested as potential anticancer, antidiabetic, and anti-inflammatory agents, and their composition was recently reported [18].

According to Euro+Med PlantBase [19], twelve *Pulicaria* taxa grow in Morocco and *Pulicaria burchardii* Hutch. subsp. *burchardii* is one of them. This taxon, endemic of Morocco, Mauritania, and Fuerteventura (Canary Islands), is one of the two subspecies of *P. burchardii* Hutch. The other, *P. burchardii* subsp. *longifolia* E. Gamal-Eldin, is endemic of Cape Verde [1].

*P. burchardii* Hutch. subsp. *burchardii* is a very rare shrub with a hemispherical shape, densely branched, with spreading lateral branches that can root in the substrate. The tomentum is short and dense, and white in branches and leaves. The leaves are alternate, linear-spatulate, 1–3 cm long. The capitula are terminal (up to 1.5 cm in diameter) with yellow ligules and florets. It is a halo-psammophilous species, although it is capable of thriving on stony soils or even with calcareous crusts, and grows in places exposed to the sea wind and forms micro-dunes around it when the ground is sandy, similar to other species in its habitat [20].

The only previous phytochemical report on this taxon concerns the isolation, from the ethanolic extract of the aerial parts of the accession from Canary Island, of seven known compounds including triterpenoids, 3*β*-hydroxytaraxaster-20-en-30-al, 11-oxo-aamyrin, vanillin, 4-hydroxybenzoic acid, scopoletin, stigmasterol, and *β*-sitosterol [21]. On the other hand, nothing has been published on its EO (PBB) or on its biological properties.

EOs represent a promising phytochemical frontier with high antiviral and antibacterial efficacy against several species. The chemical diversity of bioactive molecules in EOs makes them a new potential source of antiviral drugs, which could overcome the limitations associated with current synthetic drug treatments. In detail, it has been documented that EOs from oregano, cloves, and *Melaleuca alternifolia* have shown activity against *Herpes simplex* virus type-1 (HSV-1), adeno virus type-3, coxsackie virus B-1, and the polio virus [22,23]; phenylpropanes, sesquiterpenes, and triterpenes, components of EOs, have been found to act against HSV-1, poliovirus, and the influenza virus [24,25,26]. However, to date, no study has investigated the antiviral, antioxidant, and antibacterial potential of the essential oil derived from *P. burchardii* subsp. *burchardii*. In this context, the present investigation evaluated the efficacy of PBB against HSV-1 and SARS-CoV-2, representing viral models with DNA and RNA genomes, respectively. The analysis was also conducted at different stages of viral infection to better understand the PBB mechanism of action. Furthermore, the possible anti-germinative actions of PBB on both *B. subtilis* and the food pathogen *B. cereus* were studied to evaluate its possible use as a natural food preservative. The particular composition of PBB, essentially consisting of an abundant and primary component 1-oxo-bisabolone, a rare compound, makes it a unique and interesting candidate for many applications.

## 2. Results

### 2.1. Essential Oil Composition

Hydro-distillation of *P. burchardii* Hutch. subsp. *burchardii* flowering aerial parts gave a yellow-orange EO (PBB). Overall, twenty-seven compounds were identified in PBB, representing 92.37% of the total composition. The components are listed in Table 1 according to their retention indices on a DB-5MS column and are classified based on their chemical structures into five different classes.

**Figure 1:**
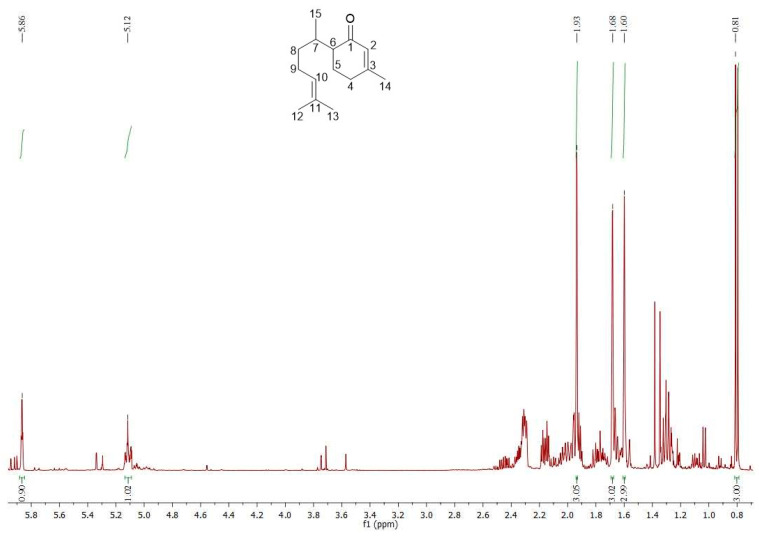
^1^H-NMR spectrum of PBB and structure of 1-oxo-bisabolone.

The PBB was extremely rich in oxygenated sesquiterpenes (72.59%), mainly represented by 1-oxo-bisabolone (65.09%), followed by a limited quantity of *τ*-cadinol (4.21%). Eucalyptol (13.87%) was the most abundant metabolite among the oxygenated monoterpenes (15.03%), whereas only *α*-pinene (2.24%) is worthy of mention among the monoterpene hydrocarbons (2.40%). The ^1^H-NMR spectrum of PBB (Figure 1) showed characteristic signals for one aliphatic methyl C*H_3_*-15 (0.81 ppm, *J* = 6.7 Hz, d, 3H), three allyl methyl groups (1.60, 1.68, and 1.93 ppm, brs, 3H × 3, for C*H_3_*-12, C*H_3_*-13, and C*H_3_*-14, respectively), and two double bonds confirmed by the presence of two signals at 5.12 (H-10, 1H) and 5.86 (H-2, 1H) ppm. Figures S1 and S2 show enlargements of the proton spectrum of PBB.

In the carbon spectrum (Figure S3), as well as confirming the presence of a clear conjugated ketone (201.07 ppm, C-1) with a double bond (161.09 and 127.15 ppm, C_3_-C_2_), it showed fifteen peaks in agreement with the data in the literature [27,28]. Table 2 shows the ^1^H- and ^13^C-NMR data of the main bisaboloid. Consequently, the main metabolite of PBB was confirmed as 1-oxo-bisabolone.

### 2.2. Antimicrobial Properties of PBB

**Figure 2:**
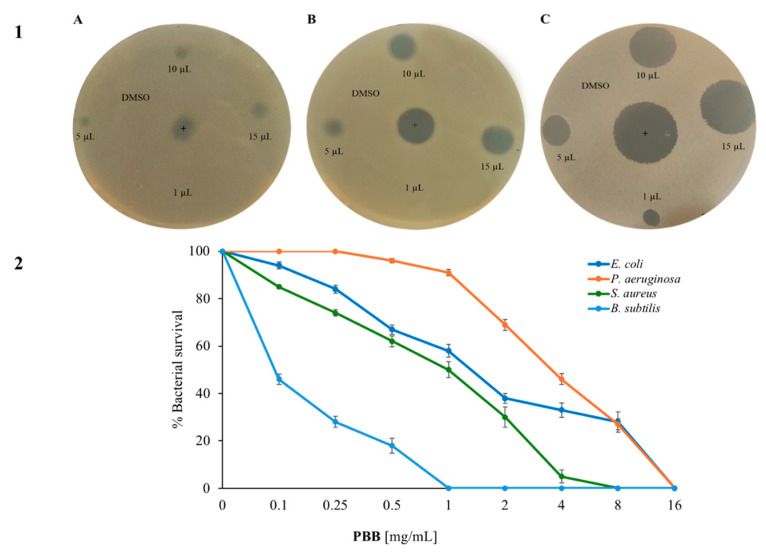
Antimicrobial activity of PBB: Subfigure 1 shows the halo of inhibition against (**A**) *E. coli*, (**B**) *S. aureus*, and (**C**) *B. subtilis*; subfigure 2 shows the activity of PBB used at different concentrations (0, 0.1, 0.25, 0.5, 1, 2, 4, 8, and 16 mg/mL) by cell counting test colonies after 4 h of incubation. This was tested against the Gram-negative bacteria *E. coli* and *P. aeruginosa*, and against the Gram-positive bacteria *S. aureus* and *B. subtilis*. The *y*-axis represents the percentage of bacterial survival. Each test was performed in three independent experiments, and the standard deviation was less than 5%.

The first assay carried was the Kirby and Bauer test. Subfigure A shows how the quantity of PBB increases as the bacterial growth decreases, and the diameter of the halo increases. It is possible to see that Gram-negative bacteria were less sensitive to PBB than the Gram-positive bacteria at the same concentrations. Among the Gram-negative, it seems that the PBB had a higher activity on *B. subtilis* than on *S. aureus*. Subsequently, the antimicrobial activity of PBB was analyzed via the cell survival test by using dose–response curves on the selected strains and increasing the concentration of PBB. Subfigure B (Figure 2) shows that PBB had strong antimicrobial activity with a dose-dependent trend. In particular, it appeared to be active at lower concentrations on Gram-positive than on Gram-negative bacteria. Furthermore, the most sensitive strain appeared to be *B. subtilis*; in fact, at a concentration of 1 mg/mL, the bacteria were completely killed.

The minimum inhibitory concentration index (MIC) of some of the Gram-positive bacteria was also calculated, as shown in Table 3.

In this case, the lowest MIC value was obtained against *B. subtilis* (0.6 mg/mL). For the other strains tested, the MIC value was higher; in fact, it was 5 mg/mL for *B. cereus*, and 6 mg/mL for *B. licheniformis*. Subsequently, it was demonstrated that the PBB was bacteriostatic on the three selected spore-forming bacilli. This was because, after re-incubating the wells corresponding to the MIC value, a turbidity comparable to the positive control was observed after 24 h, as measured by OD at 600 nm.

### 2.3. Analysis of PBB Mechanism of Action Utilizing Fluorescence Microscopy

To discern the potential mechanism of action of the active molecules present in PBB, fluorescence microscopy was used to examine the effect on the selected bacterial strains. *E. coli*, *S. aureus,* and *B. subtilis* were chosen as the indicator strains.

The bacteria were exposed to PBB at the highest concentration used in the other experiments, and two dyes were added: 4′,6-diamidino-2-phenylindole dihydrochloride (DAPI), a DNA intercalator that stains live cells with a blue color, and propidium iodide (PI), a DNA intercalator that stains dead cells red when it penetrates the compromised membrane. As shown in Figure S4, the treated cells of all three strains had the same shape and color as the control; in fact, all of the treated cells of *E. coli*, *S. aureus*, *B. subtilis* appeared blue, thus indicating the absence of damage to the cell membranes, results like those of the control. Therefore, we can state that the bacterial membrane remains intact after treatment with PBB for both Gram-positive and Gram-negative strains.

### 2.4. Evaluation of PBB Effects on Spore Germination

**Figure 3:**
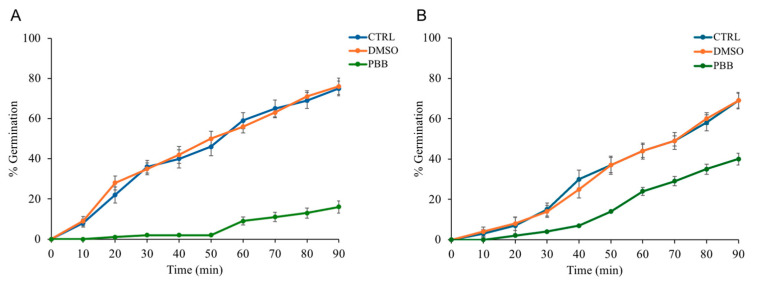
(**A**) The change in germination percentage of *B. subtilis* spores over time when exposed to PBB at 0.6 mg/mL, compared to controls (spores only, DMSO). (**B**) The germination percentage of *B. cereus* spores over time with PBB at 5 mg/mL against the same controls. Data are from three independent experiments.

The possible anti-germinative action of PBB was evaluated every 10 min for a total duration of 90 min. As illustrated by the Figure 3A showing the percentage of germination over time, compared to the controls, *B. subtilis* spores treated with PBB at a concentration of 0.6 mg/mL showed an initial block in germination, followed by a notable slowdown. Similarly, Figure 3B, depicting the germination of *B. cereus* spores treated with an PBB concentration of 5 mg/mL, also showed a deceleration in germination.

Furthermore, spores treated with DMSO (used as a solvent for the oil sample) and those without any compound showed an increase in germination percentage over time. This indicates that PBB had a significant slowdown in anti-germinative activity.

### 2.5. Antioxidant Activity of PBB

**Figure 4:**
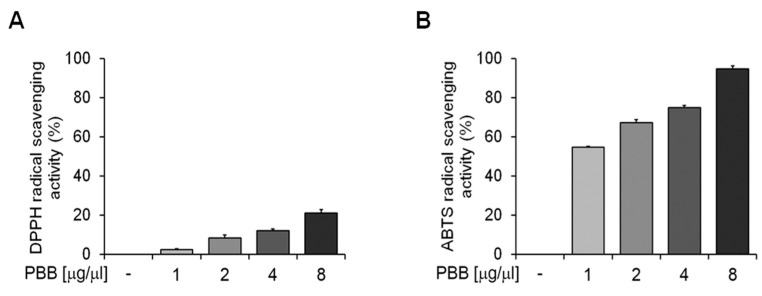
Antioxidant activity of PBB: The DPPH (**A**) and ABTS (**B**) radical scavenging activity of PBB compared to the control. Data are presented as mean values with the standard error and were analyzed using a paired t-test. Bars with different letters indicate significant differences at *p* < 0.05.

Figure 4 illustrates how increasing the concentration of PBB used (1–8 mg/mL) increased the DPPH and ABTS radical scavenging activity. Figure 4A shows the DPPH radical scavenging activity after 30 min of incubation, expressed as the percentage of DPPH removed relative to the control, while Figure 4B presents the ABTS radical scavenging activity after 10 min of incubation, shown as the percentage of ABTS removed relative to the control.

### 2.6. MTT Cytotoxic Effect of PBB in Eukaryotic Cells

**Figure 5:**
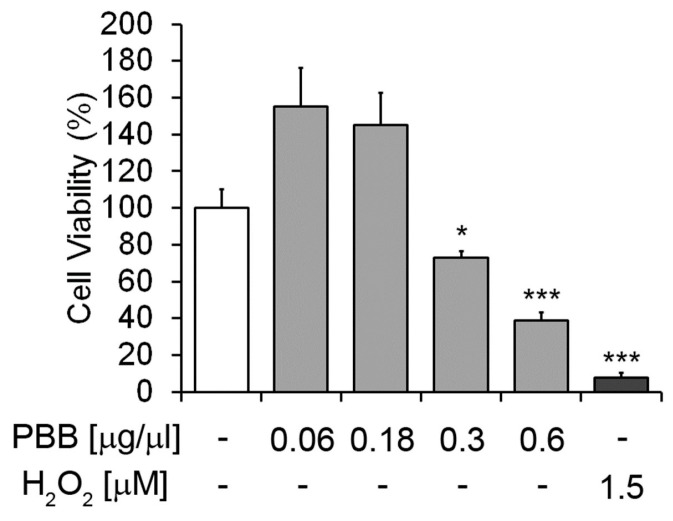
The effects of PBB on HaCat cells. Viability of the cells was assessed by the MTT assay. The untreated cells were assumed as 100%. Percent of cell survival in each condition was calculated by comparing with the untreated cells. * *p* < 0.01, *** *p* < 0.001, *n* = 3.

The cytotoxic effect of different concentrations of PBB on HaCat cells is shown in Figure 5. The figure shows that PBB significantly and concentration dependently decreased the viability of HaCat cells. A low concentration of PBB showed no significant inhibitory effects on cell viability compared to the untreated cells.

### 2.7. Cellular Toxicity Analysis

**Figure 6:**
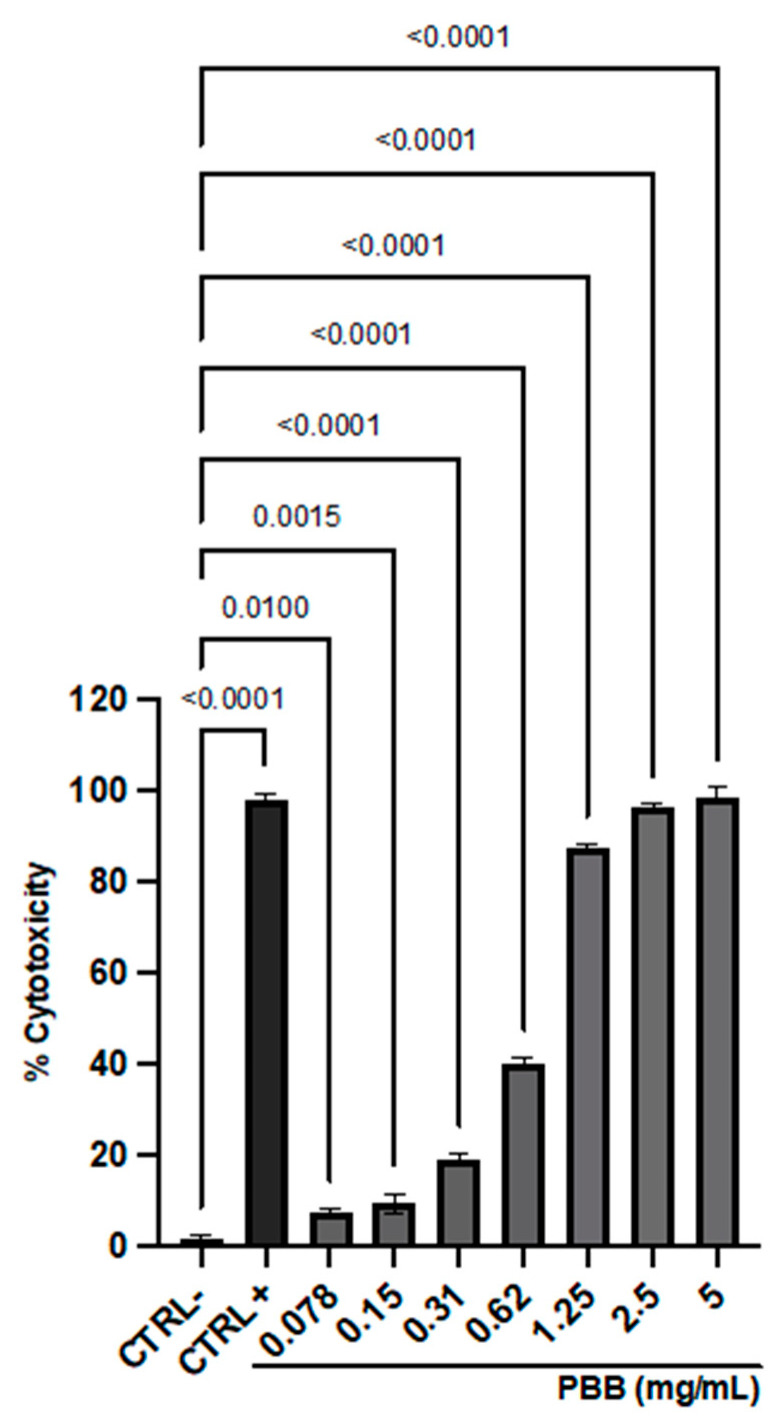
Cytotoxic effect of PBB on VERO-76 cells after 24 h exposure. Data represent the mean ± standard deviation (SD) of three independent experiments.

Cytotoxicity was assessed on VERO-76 cells via the MTT assay after 24 hour exposure to PBB (Figure 6).

The sample induced dose-dependent toxicity, with a mortality rate of 100% at the highest concentration tested and up to 1.25 mg/mL. Inversely, the cytotoxicity recorded at 0.31 mg/mL was 16%, which was subsequently selected to assess the antiviral activity. The 100% DMSO, used as the CTRL+, induced a cytotoxicity rate of 98.7%. The 50% cytotoxic concentration (CC_50_) and the 90% cytotoxic concentration (CC_90_) of PBB were 0.5971 and 2.03 mg/mL, respectively.

### 2.8. Antiviral Properties of PBB

**Figure 7:**
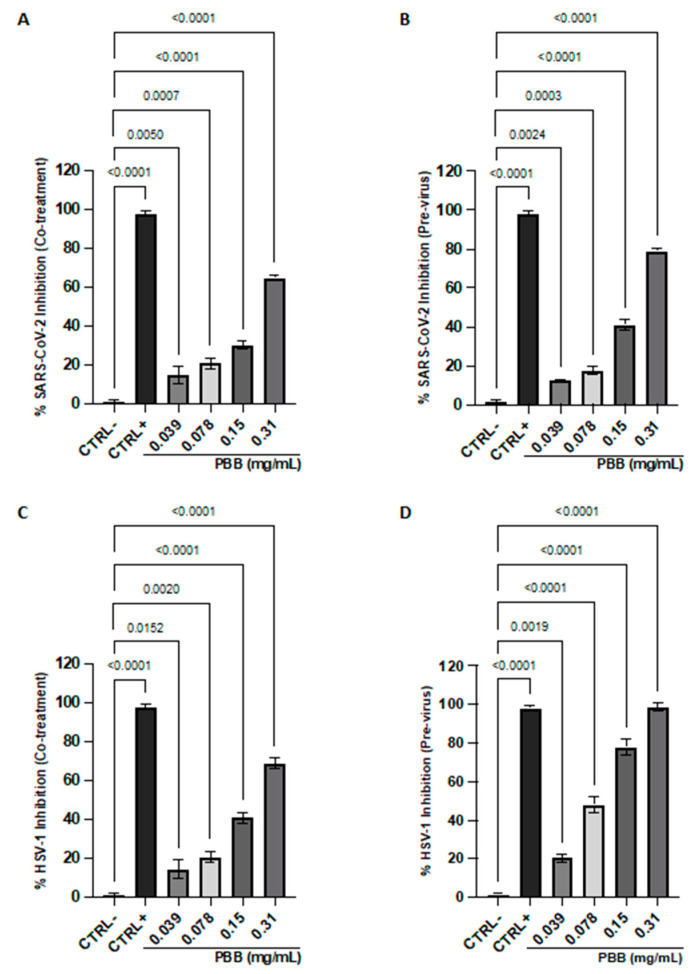
Plaque reduction assay of PBB lysis against SARS-CoV-2 (**A**,**B**) and HSV-1 (**C**,**D**). Data represent the mean ± standard deviation (SD) of three independent experiments.

The antiviral activity of PBB was investigated against SARS-CoV-2 (Figure 7A,B) and HSV-1 (Figure 7C,D).

The assays were conducted at different stages of the infection cycle: viral pretreatment, where viral particles were exposed to the extract before infecting the cell monolayer, and co-treatment, where viral particles and the oil sample were added simultaneously to the cell monolayer. Efficacy was assessed by measuring the reduction in plaque formation within the infected monolayer. The effectiveness was measured by assessing the reduction in lysis plaques in the infected monolayer. The results indicate the activity of PBB against both viral strains tested, with more pronounced effects against HSV-1 compared to SARS-CoV-2. In detail, in the co-treatment assay, at the highest sub-toxic concentration tested (0.31 mg/mL), the sample oil achieved viral inhibition rates of 69% and 65% for HSV-1 and SARS-CoV-2, respectively. Furthermore, an increase in the antiviral effect was observed in the extracellular environment, suggesting the inhibition of viral contact and fusion with the host cell. In this phase, the total inhibition of HSV-1 was recorded at 0.31 mg/mL and a 78% reduction against SARS-CoV-2, suggesting a possible virucidal effect of the oil. Conversely, no relevant inhibition was observed in the cell pre-treatment and the post-infection assays. The inhibitory concentration 50% (IC_50_) and inhibitory concentration 90% (IC_90_) values obtained in the previrus were 0.092–0.19% and 0.235–0.524% against HSV-1 and SARS-CoV-2, respectively.

## 3. Discussion

The presence of 1-oxo-bisabolone in so large an amount is quite interesting. In fact, it has been identified in a small amount (2.5%) in the EO of *Pulicaria gnaphalodes* (Vent.) Boiss. [29]. In the literature, there are quite a few papers reporting the occurrence of 1-oxo-bisabolone from a natural source. The first report of its presence (15%) in *Stevia purpurea* Pers was made by [30]. Later on, it was reported in the EOs of two Poaceae: *Cymbopogon citratus* Staff [27] and *Cymbopogon distans* (Steud.) Wats. [31]. A good quantity of 1-oxo-bisabolone was detected in the leaf EO of *Commiphora africana* (A. Rich.) Engl [28], whereas the EO of the two Asteraceae, *Asteriscus graveolens* (Forssk.) Less [32] and *Geigeria alata* (DC.) Oliv. *et* Hiern. [33], contained limited amounts. Finally, its presence in the EO of *Rhododendron thymifolium* Maxim (Ericaceae) and its insecticidal properties were recently determined [34]. Although 1-oxo-bisabolone has been detected in only one *Pulicaria* taxa, some bisabolene derivatives have been identified in the EOs of some species such as *β*-bisabolol in *P. gnaphalodes* (Vent.) Boiss. [29], *α*-bisabolol in *P. somalensis* O. Hoffm. [35], *P. undulata* (L.) C. A. Mey [36,37], and *β*-bisabolene in *P. vulgare* ssp. *graeca* (Sch.-Bip.) Fiori [38].

Specifically, 1-oxo-bisabolone is an oxygenated sesquiterpene derived from bisabolone, a compound found in many medicinal plants. For instance, chamomile is one of the most well-known medicinal plants containing sesquiterpenes including various bisabolone oxides [39]. Chamomile extracts have also demonstrated antimicrobial properties against both Gram-positive and Gram-negative bacteria. Tocai-Moțoc et al. [40] highlighted that the EO obtained from *Matricaria chamomilla* L. flowers, rich in bisabolol oxide, bisabolol, and farnesene, exhibited significant antimicrobial activity against *E. coli* and *S. aureus*.

Avonto et al. demonstrated that in extracts of German *Matricaria chamomilla*, used as a medicinal plant in Western herbal medicine, bisabolol and its oxidized metabolites served as marker compounds for distinguishing different chemotypes. These compounds contribute to the therapeutic properties, such as anti-inflammatory, antibacterial, insecticidal, and anti-ulcer, of this species [41].

Sage is another plant that contains sesquiterpenes including compounds similar to 1-oxo-bisabolone. Sage extracts have been studied for their antimicrobial properties. Paknejadi et al. demonstrated that the EOs derived from five different species of sage showed significant antimicrobial activity, particularly against Gram-positive strains such as *S. aureus* [42].

Other EOs have also demonstrated notable antimicrobial activities attributable to the presence of sesquiterpenes. Castagliuolo et al. demonstrated that the EO of *Thymus richardii* subsp. *nitidus* exhibited significant antimicrobial activity, especially against Gram-positive strains, attributable to the presence of thymol and *β*-bisabolene [43]. The results of our study are consistent with the existing literature, highlighting that 1-oxo-bisabolone is likely the primary agent responsible for the antimicrobial activity of PBB. We observed a higher susceptibility to these compounds in Gram-positive strains. Both the plate inhibition assay and the viable count assay indicated that PBB exhibited higher activity against *S. aureus* and *B. subtilis* compared to *E. coli* and *P. aeruginosa*. Since *B. subtilis* initially proved to be the most sensitive strain, we decided to further investigate the antimicrobial activity against other bacilli, such as *B. cereus* and *B. licheniformis*, by calculating the MIC. These bacilli showed surprisingly low MIC values of 6 and 5 mg/mL, respectively, with bacteriostatic potential.

The precise mechanism by which EO exerts its antimicrobial properties is not always fully understood. Considering the large number of different chemical compounds present in EOs, it is likely that their antibacterial activity is not due to a single specific mechanism, but rather to multiple cellular targets. Cena stated that EOs, in general, can destabilize bacterial cell membranes, causing the loss of cellular contents and consequently bacterial death. However, their antimicrobial activity is likely due to the presence of various molecules in their composition that work synergistically, acting on multiple target sites within the bacterial cell [44]. In our case, it was evident that the target was not the cell membranes, as the DAPI/PI double staining showed a marked blue fluorescence from DAPI, associated with a decrease in the number of cells. This indicates that the cell membranes were intact and suggests that the mechanism of action may involve other processes such as the inhibition of protein or DNA synthesis. Thus, PBB, by interfering with crucial biosynthetic processes for microbial survival, could exert its antimicrobial activity. In investigating the antibacterial mechanisms associated with *α*-bisabolol, Cruz et al. demonstrated that *α*-bisabolol is capable of inhibiting the efflux pumps TetK and NorA, responsible for pumping antibiotics into the extracellular space [45]. Unsubstituted sesquiterpenes like *α*-humulene non-specifically target the membrane of Gram-positive bacteria, increasing permeability and the loss of intracellular contents [46]. Introducing a lactone fraction into sesquiterpenes enhances their activity against filamentous fungi, as seen with costunolide, cynaropicrin, deacetylxanthumin, and isoalantolactone [47].

Various EOs, in addition to possessing antimicrobial, antioxidant, and antiviral activities, also exhibit anti-germinative properties. Sakai et al. demonstrated that the germination times of *B. subtilis* spores in a medium containing carvacrol and thymol were delayed. Specifically, the addition of carvacrol and thymol inhibited the germination of *B. subtilis* spores, with thymol exhibiting a more pronounced degree of inhibition than carvacrol [48]. PBB also showed anti-germinative capacities for the spores of *B. subtilis* and *B. cereus*. Specifically, *B. subtilis* spores treated with PBB at a concentration of 0.6 mg/mL exhibited an initial block in germination, followed by a significant slowdown compared to the controls. Similarly, the germination of *B. cereus* spores treated with a concentration of 5 mg/mL also showed a deceleration in germination.

Plant-derived substances also offer a promising avenue for the discovery of lead compounds that can guide the development of new antiviral agents [49]. Among the vast array of plant-based compounds, EOs have gained significant attention in recent years for their antimicrobial properties. These EOs, which constitute an important component of the plant’s chemical defense system, are increasingly being evaluated for their potential to combat various pathogens including viruses [50]. EOs are complex mixtures of secondary metabolites including terpenes, phenylpropanoids, and other aromatic compounds, traditionally used for their therapeutic properties [51]. Their potential as antiviral agents have been explored through numerous studies, evaluating their effectiveness in inhibiting viral replication, interrupting viral entry, and interfering with viral assembly [52]. In this context, the present study aimed to evaluate the antiviral potential of PBB against SARS-CoV-2 and HSV-1, two viruses with markedly different envelope compositions and genome organizations. This evaluation provides valuable insights into the potential of PBB as a broad-spectrum antiviral agent. To determine the range of non-toxic concentrations for the antiviral evaluation of PBB, cytotoxicity assays were conducted on VERO-76 cells using concentrations ranging from 5 mg/mL to 0.0078 mg/mL. PBB exhibited 50% cytotoxicity at a concentration of 10 mg/mL. However, at concentrations starting from 0.31 mg/mL, its cytotoxicity was less than 10%. Based on these findings, PBB was chosen for further antiviral assays. Döll-Boscardin et al. reported that the EOs of *Eucalyptus benthamii* exhibited CC_50_ values between 108.33 μg/mL and 54.96 μg/mL, respectively, on Jurkat cells. For J774A.1 cells, the CC_50_ values were 287.98 μg/mL and 252.55 μg/mL, while for Hela cells, EOs induced 50% cytotoxicity at concentrations of 84.24 μg/mL and 110.02 μg/mL, respectively [53]. Tavakoli et al. assessed the cytotoxic potential of EOs from the aerial parts of *Ferulago trifida* on the MCF7, A-549, and HT-29 cell lines. The recorded CC_50_ values were 22.0 μg/mL, 25.0 μg/mL, and 42.55 μg/mL, respectively [54]. These studies demonstrate that cytotoxicity varied not only with plant maturation, but also with the cell line used. To date, the Food and Drug Administration (FDA) has approved over 90 antiviral drugs. However, the increase in drug-resistant viral strains highlights the need for new broad-spectrum antiviral drugs. EOs have shown potential in antiviral research, offering a natural alternative or complement to traditional therapies. In this study, we report the antiviral activity of PBB against SARS-CoV-2 and HSV-1 within a concentration range of 0.31 to 0.039 μg/mL. PBB demonstrated virucidal action against HSV-1 and SARS-CoV-2, with CC_50_ values of 45% and 49%, respectively. We hypothesize that PBB alters the viral surface, thereby preventing the initial infection phases of attachment and fusion of the virus to the host cell. Several studies have documented the virucidal action of EOs, highlighting their potential as antiviral agents. Ribas Pilau et al. evaluated the antiviral activity of *Lippia graveolens* EO against HSV-1. At a concentration of 99.6 μg/mL, the EO inhibited viral infectivity by 50%. This inhibition was attributed to alterations in the viral envelope structure, which subsequently influenced the primary phase of viral infection [55]. Elaissi et al. evaluated the antiviral potential of *Eucalyptus* EO against Coxsackie virus B3. The EO demonstrated an IC_50_ value of 0.7 mg/mL in the previrus assay. This suspension also affected the attachment and fusion phases of the viral particle with the host cell [56]. Ćavar Zeljković et al. demonstrated the virucidal potential of *Mentha aquatica* EO against SARS-CoV-2, where 50% of the viral particles showed structural alteration of the viral envelope at a concentration of 189.73 mg/L [57]. Similarly, Haddad et al. reported the virucidal action of EO, and their research demonstrated that the *Ayapana triplinervis* EO, at a concentration of 38 μg/mL, reduced the infectivity of the Zika virus by 50% and effectively destroyed the viral envelope [58]. While the reported studies shared the observation of the virucidal action of EOs, the active doses were different. The latter were influenced by the composition of the EO, and above all, by the viral species studied. The compounds contained in PBB, and in particular 1-oxo-bisabolone, are very interesting and can be studied in the future for potential biological applications, hoping to lead to the development of new drugs or pesticides.

## 4. Materials and Methods

### 4.1. Plant Materials

The fresh aerial parts (stems and leaves) of *Pulicaria burchardii* Hutch. subsp. *burchardii* were collected near Chichaoua (Morocco), (31°32’53” N; 8°48’39” E 378 m.s.l.), in May 2023. A voucher of this plant, identified by Prof. Ilardi, was deposited at the herbarium in the university complex of Palermo (Vouchers No. PAL 109774).

### 4.2. Isolation of Volatile Components and Spectroscopical Analyses

For the extraction of the EO, the procedure reported in Lauricella et al. [59] was performed. The aerial parts, freshly ground, were subjected to hydrodistillation using the Clevenger apparatus following the procedural indications of the European Pharmacopoeia [60]. Once the EO was obtained, it was dried and stored at −20 °C until analysis; the sample yield was 0.1% (*w/w*). The NMR spectra were acquired with a Bruker Avance II instrument (400 MHz for ^1^H-NMR and 100 MHz for ^13^C-NMR) [61] using chloroform (CDCl_3_) as the deuterated solvent with 7.27 and 77.0 ppm as the peak references for the H- and C-NMR spectra, respectively. *δ*_C_ (ppm) 15.57 (C-15), 17.62 (C-12), 22.35 (C-5), 24.08 (C-14), 25.68 (C-13), 25.99 (C-9), 30.26 (C-7), 30.89 (C-4), 34.69 (C-8), 49.84 (C-6), 124.90 (C-10), 127.15 (C-2), 131.38 (C-11), 161.09 (C-3), 201.07 (C-1).

### 4.3. GC and GCMS Analyses

Analysis of PBB was performed according to the procedure reported by Gagliano Candela et al. [62].

### 4.4. Bacterial Strains

The antimicrobial effect was assessed using the following bacterial strains. Gram-negative: *Escherichia coli* DH5α and *Pseudomonas aeruginosa* PAOI ATCC 15692; Gram-positive: *Staphylococcus aureus* ATCC6538P, *Bacillus subtilis* AZ54, *Bacillus cereus* ATCC10987, and *Bacillus licheniformis* DSM8782.

### 4.5. Antimicrobial Assay

The presence of potential antimicrobial compounds in the essential oil of *P. burchardii* was investigated using the agar diffusion method, based on the Kirby–Bauer protocol with some modifications [63].

Three different volumes (5, 10, and 15 µL) of PBB were placed on Luria–Bertani agar plates overlaid with 10 mL of soft agar (0.7%) and pre-mixed with 10 μL (5 × 10^5^ CFU/mL) of *E. coli* DH5α, *S. aureus* ATCC6538P, and *B. subtilis* AZ54 grown for 24 h at 37 °C. The negative control was represented by dimethyl sulfoxide 80% (DMSO) used to resuspend the samples; the positive control was the antibiotic ampicillin (10 µL) concentrated at 0.1 mg/mL. After incubation at 37 °C, antimicrobial activity was calculated based on the diameter of the inhibition zone as arbitrary units per milliliter, according to the equation shown by Bhaskar et al. [64].

Another method to evaluate antimicrobial activity involved counting the cell viability of the Gram-positive and Gram-negative strains. The bacterial cells (5 × 10^5^ CFU/mL) were incubated with PBB at a concentration of 0.1, 0.25, 0.5, 1, 2, 4, 8, and 16 mg/mL. The percentage of bacterial survival was calculated following the protocol shown by Castagliuolo et al. [43]. Each experiment was conducted in triplicate and the results reported were an average of three independent experiments.

### 4.6. Determination of Minimal Inhibitory Concentration

The minimum inhibitory concentrations (MICs) of PBB against all bacterial strains were determined using the microdilution method as outlined by the Clinical and Laboratory Standards Institute (CLSI). In brief, ~5 × 10^5^ CFU/mL was added to 95 µL Mueller-–Hinton broth (CAM-HB; Difco) with or without PBB at different concentrations (0.1–16 mg/mL). Following overnight incubation at 37 °C, the MIC_100_ values were identified as the lowest concentration at which no visible bacterial growth was observed. After that, 5 µL of each sample at the MIC value was subsequently added to 95 µL of fresh medium in 96-well plates and re-incubated at 37 °C for 24 h. Subsequently, we evaluated whether the antimicrobial activity of PBB was bacteriostatic or bactericidal by reading the OD at 600 nm. If there was bacterial growth, the action was bacteriostatic, and if there was no growth, the action was bactericidal. All experiments were conducted in triplicate, and the results were presented as the average of three independent trials.

### 4.7. Fluorescence Microscopy Experiments: DAPI/PI

For the fluorescence microscopy experiments, two dyes were used: DAPI (4’,6-diamidino-2-phenylindole dihydrochloride; Sigma Aldrich, Milan, Italy) and IP (propidium iodide; Sigma Aldrich, Milan, Italy). Briefly, 100 µL of bacterial cultures of *E. coli* DH5α, *S. aureus* ATCC6538P, and *Bacillus subtilis* (grown to mid-log phase) were incubated in the dark for 2 h at 37 °C with shaking, with or without PBB. After incubation, 10 µL of the bacterial culture was mixed with a solution of DAPI [1 µg/mL] and PI [20 µg/mL]. Samples were examined with an Olympus BX51 fluorescence microscope (Olympus, Tokyo, Japan) equipped with a DAPI filter (excitation/emission: 358/461 nm). Standard acquisition times for DAPI/PI dual staining were set at 1000 ms. Images were acquired using an Olympus DP70 digital camera, following the method described by Pizzo et al. [65].

### 4.8. Spore Production and Purification

Sporulation of *B. subtilis* was induced using the nutrient depletion method [66]. In summary, after 30 h of growth at 37 °C with vigorous shaking in Difco sporulation medium (DSM) (composition per 1 L: 8 g Nutrient Broth, 1 g KCl, 1 mM MgSO_4_, 1 mM Ca(NO_3_)_2_, 10 μM MnCl_2_, 1 μM FeSO_4_, from Sigma-Aldrich, Germany), spores were observed under an optical microscope, washed four times with cold, sterile distilled water, and centrifuged at 8000× *g* for 20 min. The spore purification process involved treatment with 1 M KCl, 10 mM lysozyme, 1 M NaCl, and 0.05% SDS, followed by several water washes [67].

For *B. cereus*, sporulation was promoted by inoculating the strain into DSM sporulation medium and incubating at 25 °C with vigorous shaking for 72 h. Mature spores were washed four times with cold, sterile distilled water and further purified by incubating in H_2_O at 4 °C overnight to lyse any remaining sporangial cells. The purity of both *B. subtilis* and *B. cereus* spore preparations was confirmed by microscopy, with samples considered pure when the sporangial cells comprised less than 5% of the observed content.

### 4.9. Spore Germination Assay

The initial A 600 nm of both *B. subtilis* and *B. cereus* spore suspensions was 0.3, corresponding to approximately 4 × 10^7^ spores mL^−1^.

Purified *B. subtilis* spores were activated by heat (20 min at 70 °C) and diluted in 10 mM Tris-HCl buffer (pH 8.0) containing 1 mM glucose, 1 mM fructose, and 10 mM KCl (GFK) in the presence or absence of PBB at a concentration of 0.6 mg/mL. Following activation, germination was initiated by introducing 10 mM asparagine into the mixture, and the optical density at 600 nm was recorded every 10 min over a 60 min period [68].

Germination of *B. cereus* spores was induced by heat (70 °C for 20 min) in a germination buffer of 10 mM Tris–HCl at pH 7.4, 10 mM NaCl [69] in the presence or not of PBB at a concentration of 5 mg/mL, and subsequently supplemented with 10 mM L-alanine. The drop rate of A 600 nm was measured at 10 min intervals for 90 min.

As further controls, the germination of *B. subtilis* and *B. cereus* spores was evaluated in the presence of DMSO as the negative control and PBB as the positive control.

### 4.10. ABTS Assay

This assay, which evaluates the ABTS radical scavenging activity, was carried out following the protocol outlined by Napolitano et al., with some modifications [70]. Specifically, 1 mL of ABTS solution was mixed with 100 µL of PBB at concentrations of 1, 2, 4, and 8 mg/mL. Absorbance was measured at 734 nm against a blank, and the percentage of ABTS radical inhibition was calculated using the formula presented in their study. Each experiment was performed in triplicate and the result reported was an average of three independent experiments.

### 4.11. DPPH Radical Scavenging Assay

The DPPH (2,2-diphenylpicrylhydrazyl hydrate) radical scavenging activity was assessed as described in the literature [71]. Various concentrations of PBB (1, 2, 4, and 8 mg/mL) were prepared in 100% methanol. Freshly prepared DPPH solution (0.1 mM) was added to the PBB to a final volume of 1 mL, ensuring that the initial absorbance of the DPPH solution was ≤1.0. The reaction mixtures were incubated at 25 °C for 30 min in the dark. Absorbance was measured at 517 nm using a spectrophotometer. The scavenging activity was calculated using the equation: DPPH radical scavenging activity (%) = (1 *−* AS/AC) × 100, where AS is the absorbance of the reacted mixture of DPPH with the extract sample, and AC is the absorbance of the DPPH solution.

### 4.12. Cell Viability Assay

The MTT-based cytotoxicity assay of PBB in the HaCat cell lines was determined by a rapid colorimetric assay. In this assay, 20,000 cells were seeded in 96-well microplates and incubated for 24 h at 37 °C, 5% CO_2_ and humidified air. Then, different concentrations of PBB were added to each well. For the positive and negative controls, H_2_O_2_ (1.5 µM) and DMSO were used, respectively. The plates were incubated for 24 h in the same condition. To assess the cell survival, 10 µL of MTT in 90 µL of DMEM solution was added to each well, and the plates were incubated at 37 °C for 4 h. Then, the media were removed and 100 µL of DMSO was added to each well and incubated for 10 min. Absorbance was measured at 570 nm using a Synergy H4 Hybrid Microplate reader (Agilent, Santa Clara, CA, USA). Each concentration was assayed in four wells and repeated three times. Cell viability was calculated based on the untreated cells (DMSO) that were assumed as 100%.

### 4.13. Cytotoxicity Assessment

The 3-(4,5-dimethylthiazol-2-yl)-2,5-diphenyl tetrazolium bromide (MTT) assay was performed to evaluate the PBB cytotoxicity after exposure to the African Green Monkey (*Cercopithecus aethiops*, VERO-76) cell line. Cells were seeded and cultured in Dulbecco’s modified Eagle medium (DMEM; Gibco; Thermo Fisher Scientific, Waltham, MA, USA), adding 2 mM L-glutamine, 100 IU/mL penicillin-streptomycin solution, 4.5 g/L glucose, and 10% fetal bovine serum (FBS; Gibco; Thermo Fisher Scientific, Waltham, MA, USA). The assay was performed in a 96-well plate, and incubated at 37 °C in a humidified environment with 5% CO_2_ for 24 h. Then, cells were exposed to increasing concentrations of PBB (0.078–5 mg/mL) for 24 h. The solvent in which the compound was dissolved (1% DMSO) and 100% DMSO served as the negative (CTRL-) and positive (CTRL+) controls, respectively. After exposure, the compound/medium mixture was removed and each well was treated with MTT solution (Sigma-Aldrich, St. Louis, MI, USA) (0.3 mg/mL) for 3 h at 37 °C. A volume of 100 μL of 100% DMSO was added to allow for formazan crystal solubilization and the absorbance at 570 nm was measured by a microplate reader (Tecan, Männedorf, Switzerland) to calculate the percent cytotoxicity as follows:

(1) $$\%\;cytotoxicity=100-[100\times (\frac{OD570\;nm\;of\;the\;test\;sample}{OD570\;nm\;of\;CTR-})]$$

### 4.14. Antiviral Activity

The antiviral properties of PBB were investigated against HSV-1 (enveloped virus with DNA genome) and SARS-CoV-2 (enveloped virus with RNA genome). Both viruses were propagated and titrated by exposing the virus to VERO-76 cells at a multiplicity of infection (MOI) of 0.01. The plaque reduction assay was conducted in different conditions: co-treatment, virus pre-treatment, cell pre-treatment, and post-treatment. The assays were performed in a 12-well plate with the cells seeded at 2 × 10^5^ cells/well for 24 h at 37 °C with 5% CO_2_ and treated as follows:

1. Co-treatment: PBB in the concentration range of 0.039–0.31 mg/mL was co-exposed to the cell monolayer with the viral suspension at 2 × 103 plaque-forming units (PFU)/mL for 1 h at 37 °C;
2. Virus pretreatment: The viral suspension at 2 × 104 PFU/mL was incubated with the compound in the same concentration range for 1 h at 37 °C. Then, a 1 to 10 dilution of the mixture was performed, and the cell monolayer was infected for 1 h at 37 °C;
3. Cell pretreatment: The cell monolayer, prior exposed to the sample for 1 h at 37 °C, was then infected for another hour with the viral suspension at 2 × 103 PFU/mL;
4. Post-treatment: Cells first infected with the virus for 1 h at 37 °C were then exposed for another hour to PBB.

After the conditions described, cells were covered with a culture medium supplemented with 5% carboxymethylcellulose to limit the infection spread: 24 h for SARS-CoV-2 and 48 h for HSV-1. To evaluate the cytopathic effect, cells were fixed with 4% formaldehyde (Sigma-Aldrich, St. Louis, MO, USA) and stained with 0.5% crystal violet (Sigma-Aldrich, St. Louis, MO, USA). Plaques were counted and correlated with those coinciding with CTRL− to obtain the percentage of viral inhibition according to the following formula:

(2) $$\%\;viral\;inhibition=100-[100\times \left(\frac{plaques\;counted\;in\;the\;test\;sample}{plaques\;counted\;in\;the\;CTRL-}\right)]$$

For the CTRL+, HSV-1 melittin (5 µM) was used in the co-treatment and in the virus pre-treatment, dextran sulfate (1 µM) in the cell pre-treatment, and acyclovir (5 µM) in the post-treatment, while unexposed infected cells represented the CTRL−. For SARS-CoV-2, PBB (10 μg/mL) was used in the co-treatment and pre-treatment of the virus, ivermectin (10 μM) in the cellular pre-treatment, and remdesivir (10 μM) in the post-treatment, while unexposed infected cells represented the CTRL−.

### 4.15. Statistical Analysis

Cytotoxicity and antiviral tests were performed in biological duplicates and the results were reported by calculating the mean and standard deviation (SD). Ordinary one-way ANOVA, Dunnett’s multiple comparison test, the cytotoxic concentration at 50% (CC_50_), and inhibitory concentration at 50% and 90% (IC_50_–IC_90_) were calculated using GraphPad Prism software ver. 9 for macOS (GraphPad Software, San Diego, CA, USA, www.graphpad.com, accessed 9 January 2024). Dunnett’s multiple comparison tests expressed the significance of differences between the PBB-exposed samples compared with the unexposed samples (CTRL−). Values were considered significant at a *p*-value < 0.05. Cytotoxicity assays on the human cells were conducted in triplicate (*n* = 3), and statistical analysis was calculated using two-tailed paired t-test (* *p* < 0.01, *** *p* < 0.001).

## 5. Conclusions

This exploratory study revealed the therapeutic potential of *Pulicaria burchardii Hutch.* subsp. *burchardii* essential oil. This sample, investigated using chromatographic (GCMS) and spectroscopic (NMR) techniques, was mainly made up (65.09%) of an oxygenated sesquiterpene such as 1-oxo-bisabolone. Concurrently, the antimicrobial, anti-germinative, antioxidant, and antiviral properties were investigated. The antimicrobial activity, which does not act directly on bacterial membranes, was evaluated on several bacteria, both Gram-positive and Gram-negative, such as *E. coli*, *P. aeruginosa, S. aureus, B. subtilis*, *B. cereus*, and *B. licheniformis,* showing the highest activity against the *B. subtilis* strain (MIC 0.6 mg/mL). The antibacterial capabilities against the *Bacillus* genus were also confirmed by the already excellent anti-germinating properties at the 0.6 mg/mL dose with a deceleration in germination confirmed for up to 90 min of treatment against *B. subtilis* bacteria. For antiviral activity, the essential oil was tested against some pathogenic viruses including SARS-CoV-2 and HSV-1. In the co-treatment assay, at the highest sub-toxic concentration tested (0.31 mg/mL), the essential oil achieved viral inhibition rates of 69% and 65% for HSV-1 and SARS-CoV-2, respectively, and showed antiviral effects in the extracellular environment, suggesting the inhibition of viral contact and fusion with the host cell. In this phase, the total inhibition of HSV-1 was recorded at 0.31 mg/mL and a 78% reduction against SARS-CoV-2, suggesting a possible virucidal effect of the essential oil. The marked antibacterial and antiviral effects suggest, in a future perspective, an in-depth analysis of 1-oxo-bisabolone, to confirm all the observations made thus far.

## Figures and Tables

**Table 1:** Chemical composition (%) of *P. burchardii* Hutch. essential oil (PBB).

No.	Compound ^a^	LRI ^b^	LRI ^c^	Area (%) ^d^
1	Hexanal	805	801	0.04
2	Methyl *β*-methylvalerate	861	866	0.02
3	*α*-Pinene	932	934	2.24
4	Camphene	955	954	0.02
5	*β*-Pinene	1034	1039	0.14
6	Benzacetaldehyde	1024	1025	0.05
7	Eucalyptol	1031	1037	13.87
8	Nonanal	1102	1105	0.09
9	Camphenol	1109	1110	0.03
10	*trans*-Pinocarveol	1134	1139	0.08
11	*cis*-Verbenol	1148	1144	0.06
12	Myrtenol	1195	1198	0.35
13	Verbenyl acetate	1291	1294	0.42
14	Myrtenyl acetate	1318	1322	0.16
15	2,3-Dihydro-1,1,5,6-tetramethyl-1H-indene	1320	1325	0.07
16	Dihydro-*β*-ionone	1416	1414	0.06
17	Humulene	1449	1456	0.86
18	*γ*-Muurolene	1472	1477	0.13
19	Germacrene D	1478	1480	0.26
20	*β*-Himachalene	1493	1498	0.31
21	*β*-Curcumene	1511	1513	0.15
22	*δ*-Cadinene	1521	1525	0.31
23	Caryophyllene oxide	1593	1594	1.91
24	*τ*-Cadinol	1637	1642	4.21
25	*β*-Eudesmol	1649	1648	1.35
26	1-Oxo-bisabolone	1747	1750	65.09
27	Ethyl palmitate	1971	1975	0.09
	Monoterpene hydrocarbons			2.40
	Oxygenated monoterpenes			15.03
	Sesquiterpene hydrocarbons			2.02
	Oxygenated sesquiterpenes			72.59
	Other compounds			0.36
	Total composition			92.37

^a^ Compounds are classified in order of linear retention time on the non-polar column (DB-5MS); ^b^ Experimental LRIs on a DB-5MS non-polar column; ^c^ LRIs on DB-5 MS column reported in the literature; ^d^ Area is the peak volume percentage of the compound in the essential oil sample.

**Table 2:** ^1^H- and ^13^C-NMR data of 1-oxo-bisabolone.

Position	*δ*_C_	*δ*_H_ (*J* in Hz)
1	201.07	-
2	127.15	5.86 (q, 1.0 Hz)
3	161.09	-
4	30.89	2.30 (m) 2.30 (m)
5	22.35	1.76 (m) 1.91 (m)
6	49.84	2.15 (ddd, 12.3, 4.1, 4.1 Hz)
7	30.26	2.34 (m)
8	34.69	1.28 (m) 1.30 (m)
9	25.99	1.97 (m) 2.02 (m)
10	124.50	5.12 (tq, 7.0, 1.5 Hz)
11	131.38	-
12	17.62	1.60 (brs)
13	25.68	1.68 (brs)
14	24.08	1.93 (s)
15	15.57	0.79 (d, 6.9 Hz)

**Table 3:** Determination of the minimal inhibitory concentration (MIC) of PBB against spore-forming bacilli strains. These values were obtained from a minimum of three independent experiments.

Strains	MIC_100_ [mg/mL]
*B. subtilis*	0.6
*B. cereus*	5
*B. licheniformis*	6

## Data Availability

The data supporting the findings of this study are accessible upon reasonable request from the corresponding author.

## Supplementary Materials

The following supporting information can be downloaded at: https://www.mdpi.com/article/10.3390/plants14010068/s1, Figure S1: Enlargement of the proton spectrum between 4.80 and 6.00 ppm; Figure S2: Enlargement of the proton spectrum between 0.40 and 2.80 ppm; Figure S3: ^13^C-NMR spectrum of PBB; Figure S4: Evaluation of the antimicrobial mechanism of action of PBB, by fluorescence microscopy. The subfigures display bacterial cells of *E. coli* (A–D), *S. aureus* (E–H), and *B. subtilis* (I–N). Subfigures A, B, I, F, I, L show the cells as seen under an optical microscope, while subfigures C, D, G, H, M, N display them under a fluorescence microscope. Untreated bacterial cells are shown in subfigures A, C, E, G, I, N; cells treated with PBB are shown in subfigures B, D, F, H, L, N. All scale bars represent 1 µm (A–N).
