# Supplementary material for: Spectroscopic Characterization and Biological Effects of 1-Oxo-bisabolone-rich Pulicaria burchardii Hutch. subsp. burchardii Essential Oil Against Viruses, Bacteria, and Spore Germination
Source: Plants (Basel). 2024 Dec 29;14(1):68. doi: 10.3390/plants14010068 (PMC11723130; doi:10.3390/plants14010068)
Supplement: Supplementary file 1 [file plants-14-00068-s001.zip › plants-3376834-supplementary.pdf]

Supporting Material

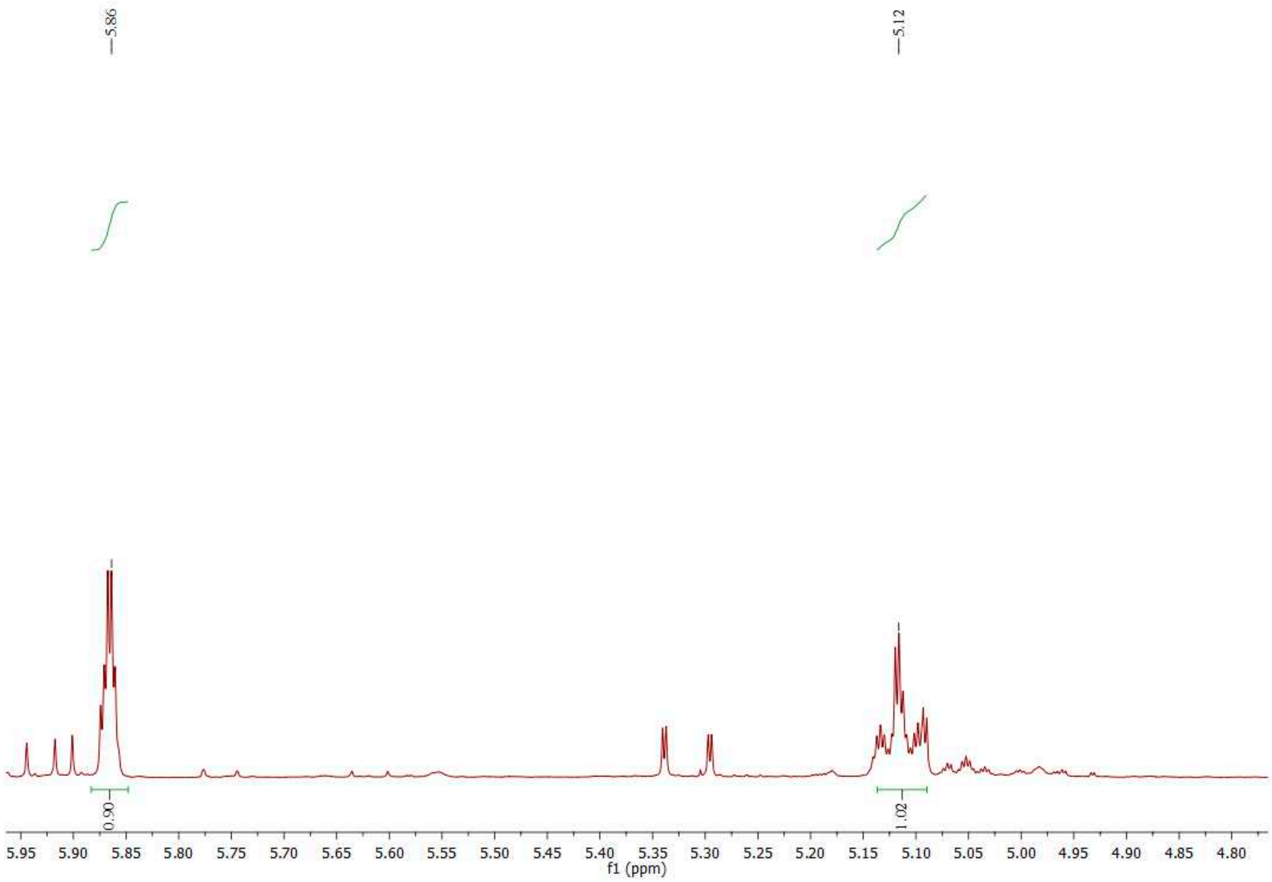

Figure S1. Enlargement of the proton spectrum between 4.80 and 6.00 ppm.

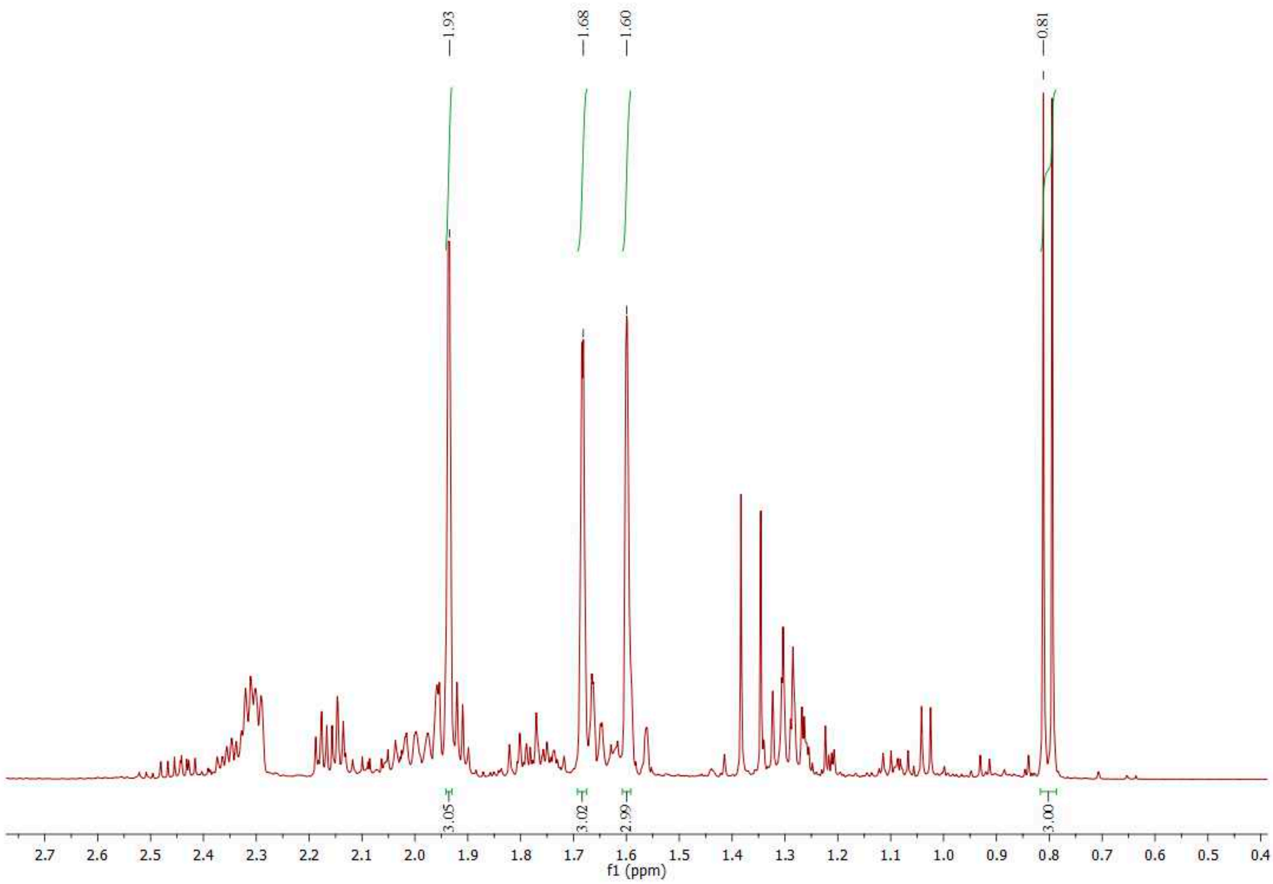

Figure S2. Enlargement of the proton spectrum between 0.40 and 2.80 ppm.

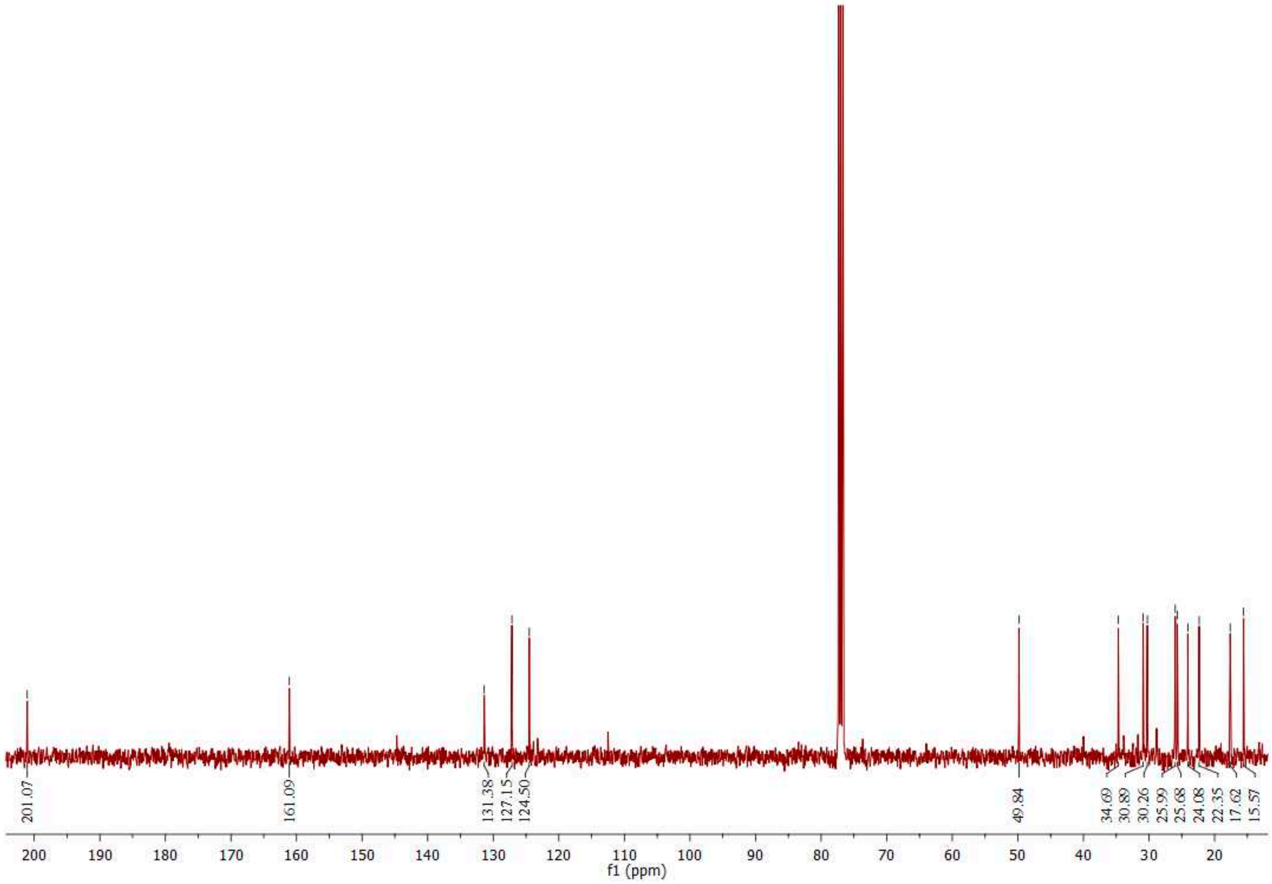

Figure S3. ^{13}C-NMR spectrum of PBB.

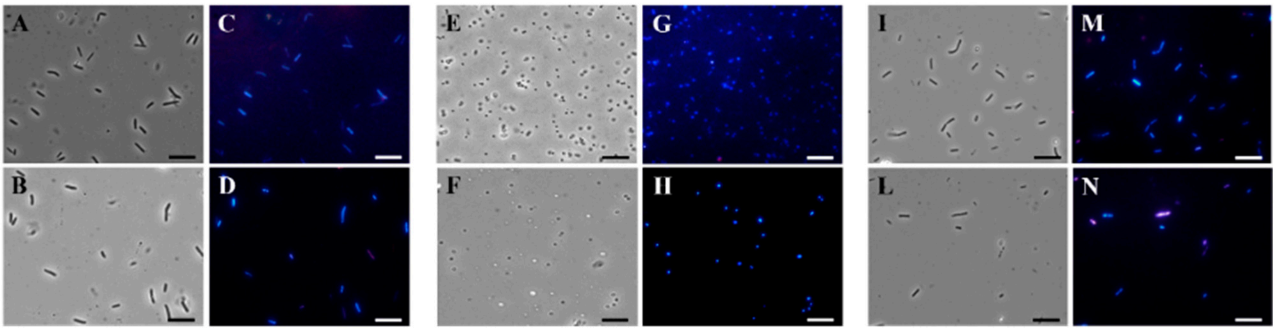

Figure S4. Evaluation of the antimicrobial mechanism of action of PBB, by fluorescence microscopy. The panels display bacterial cells of *E. coli* (A–D), *S. aureus* (E–H), and *B. subtilis* (I–N). Panels (A, B, I, F, I, L) show the cells as seen under the optical microscope, while panels (C, D, G, H, M, N) display them under the fluorescence microscope. Untreated bacterial cells are shown in A, C, E, G, I, N panels; cells treated with PBB are shown in B, D, F, H, L, N panels. All scale bars represent 1 μm (A–N).
